# Developing a method to measure bioavailable phosphorus in river water via simultaneous multisample ultrasonic extraction

**DOI:** 10.1007/s11356-024-34076-1

**Published:** 2024-06-29

**Authors:** Ting Ka Ling, Nguyen Tan Phong, Kuriko Yokota, Takanobu Inoue, Nguyen Minh Ngoc

**Affiliations:** 1 Nippon Koei Co., Ltd., 5-4 Kojimachi, Chiyoda, Tokyo 102-8539 Japan; 2 Toyohashi University of Technology, Toyohashi, Aichi 441-8580 Japan

**Keywords:** NaOH extraction, Ultrasonic washing machine, Dissolved bioavailable phosphorus, Particulate bioavailable phosphorus, Suspended sediment

## Abstract

To reduce aquatic eutrophication, measurements of bioavailable phosphorus (BAP) rather than total phosphorus (TP) are deemed critical. However, current methods require much time to separate sediments from river water, which limits the routine measurement of BAP in rivers. Therefore, in this study, a simultaneous multisample ultrasonic extraction method is proposed to directly measure total BAP (TBAP) in river water without the separation of sediment and water. Spike-and-recovery assessments showed that at least three extractions are required to maintain efficiency. A process including 2-min extraction time and three extractions was suggested. The concentrations of TBAP extracted by this process showed no significant differences with the spike calculations. Furthermore, river water TBAP was quantified using the conventional and proposed method to examine the practicality of using the proposed method for simultaneous multisample ultrasonic extraction and to evaluate its adaptability to actual river water analysis. The extracted concentrations matched those obtained using the conventional method, in which total BAP is calculated as the sum of dissolved BAP and particulate BAP; no significant difference was observed between the concentrations. Ultrasonic extraction was considerably less time-consuming than the conventional method because more samples could be analyzed during a single run. Therefore, the simultaneous multisample ultrasonic extraction method proposed in this study can be used to directly quantify total BAP in river water.

## Introduction

Nutrients such as nitrogen (N) and phosphorus (P) can enter aquatic ecosystems, and excessive amounts can lead to eutrophication and deterioration of the ecosystem health of rivers, lakes, and enclosed coastal areas (Dupas et al. 2015; Liu et al. 2017). Eutrophication has emerged as a global problem because it leads to the excessive growth of phytoplankton in aquatic systems, which in turn results in algal blooms (Diaz and Rosenberg 2008). Algal proliferation is destructive to typical ecosystems, and algal biomass degradation by bacteria leads to oxygen consumption, resulting in hypoxia (Kennish 2002). Therefore, N and P management should be monitored properly in eutrophication management strategies to mitigate this problem. In freshwater systems, because P has been the limiting factor for algal growth more frequently than N (Schindler 1977, Hecky and Kilham 1988, Elser et al. 2007), assessment of P forms, which are loading to eutrophication, is significant to freshwater environment management.

To assess environmental health and to manage eutrophication, total phosphorus (TP) has been widely monitored (Ekholm and Krogerus 2003). The standard method for TP analysis and determination involves the oxidative digestion of river water samples to decompose organic, polymer, and colloidal P substances into reactive forms, which are then analyzed via the molybdenum blue method (Murphy and Riley 1962). The most commonly used oxidant for P digestion is potassium peroxodisulfate, which is classified as a mild oxidant (Menzel and Corwin 1965). Although TP has been a common parameter in routine water quality monitoring programs, its reliability as a eutrophication indicator has been recently questioned (Boström et al. 1988, Gerdes and Kunst 1998; Pacini and Gächter 1999; Ellison and Brett 2006). TP covers the entire range of P species, including complex forms, such as carbonate and apatite P, humic P, and refractory mineral P colloidal P substances, which are not available for algal growth. The P fraction which grows algal blooms contributing to eutrophication has been defined as bioavailable phosphorus (BAP) (Ellison and Brett 2006).

Regardless of many chemical and physical forms, TP can be operationally divided into dissolved phosphorus (DP) and particulate phosphorus (PP). DP and PP are separated by filtration or centrifugation. DP is mostly available for algal growth, while PP is partially bioavailable (Ellison and Brett 2006). The percentage of PP that is bioavailable varies greatly depending on river flow characteristics and surrounding land use impacts (Ellison and Brett 2006). When river flow is high during rainfalls or storm events, PP associated with soil particles is transported to rivers, attached to suspended sediment (SS) (Meyer and Likens 1979; Uusitalo 2000; Ellison and Brett 2006). If land is used for agriculture and P-containing fertilizers are applied, the bioavailability of PP may be high. When river flow is at around base flow during periods of little or no rainfall, there are no PP land runoffs, and the bioavailability of PP is influenced by the composition of SS in rivers. If P is loosely adsorbed on SS surfaces, or it is bound to clays, easily degradable organic matters, redox-sensitive Fe and Mn, and hydroxide exchangeable Al oxides, the PP may be bioavailable. Then, more bioavailability will be released from PP when the river flow increases or human-induced disturbance in land use stirs up sediments. The dramatic fluctuations in the bioavailability of PP have attracted researchers’ attention to measure particulate bioavailable phosphorus (PBAP) separately from dissolved bioavailable phosphorus (DBAP). The sum of PBAP and DBAP is being used to determine total bioavailable phosphorus (TBAP) in a water environment (Boström et al. 1988, Sharpley et al. 1992, Ellison and Brett 2006, Rönspieß et al. 2021).

Recent reports have suggested biological and chemical methods to measure PBAP, but these methods are time-consuming. Among biological methods, algal growth potential (AGP) is the most direct method used for estimating the bioavailability of P in samples (Miller et al. 1980; Raschke and Schultz 1987; Klapwijk et al. 1989; Okubo et al. 2012). The AGP test requires a 14-day incubation; thus, it is not suitable for quantifying BAP in a large number of samples (Schultz et al. 1994). Faster chemical methods have been developed; the most common method using 0.1 M NaOH as an extractant involving single extraction through mechanical shaking was suggested by Sharpley et al. (1991). NaOH can extract loosely adsorbed P, clay-bound P, redox-sensitive Fe, Mn, and exchangeable Al-bound P (Sharpley et al. 1991; Pacini and Gächter 1999). Previous studies have shown the P extracted with 0.1 M NaOH could be uptaken by algae (Williams et al. 1980; Fabre et al. 1996). This method is often used to measure BAP, even though it takes 17 h for the extraction process, and another day to complete concentration analyses. Therefore, we considered ultrasonication to fasten the extraction process.

Ultrasonication has been used to extract various elements from food and environmental samples because it is an efficient technique that requires less time than previously used methods, improving sampling yields. Ultrasonic extraction is based on the principle of acoustic cavitation, during which energy is transferred to attached substances in the extractant (Chemat et al. 2011). Consequently, it requires less time and is more efficient (Song et al. 2015). An ultrasonic extraction method with an extraction time of 1 min using an ultrasonic horn was developed for estimating the BAP of particulate matter (Ngoc et al. 2017a, b). Even so, the problem is that this method can only be used for the extraction of one sample at a time, making simultaneous multisample extraction impossible. Since the number of samples that can be estimated using this method is limited, it is unsuitable for use in routine laboratory analysis. Therefore, it is necessary to develop a new method for BAP measurement that requires less time and is able to provide a higher level of efficiency than the conventional method. Ultrasonic washing machine is a promising instrument. Ultrasonic washing machines have commonly been used to clean medical devices, electronics, and experimental laboratory apparatuses because they can produce cavitation to rapidly and completely remove dirt and debris (Niemczewski 2009). Previous studies have shown that ultrasonic washing machines could be used to extract chemical substances from plants and foods in short time periods (Chemat et al. 2011). Additionally, previous studies have also proven that ultrasonic washing machines can be used for extracting particulate bioavailable phosphorus (PBAP), and no significant difference was found compared with the conventional method (Ka Ling et al. 2021). Hence, this study focused on developing a simultaneous multisample extraction method using an ultrasonic washing machine to extract TBAP from river water without separating PBAP and DBAP.

Although ultrasonic treatment has potential, its application for the quantification of TBAP is still uncertain. Accordingly, this study’s objective was to develop a simple method that can quantify TBAP directly from river water both quickly and accurately. Therefore, the following aims were considered.

- To examine the DBAP extraction efficacy of an ultrasonic washing machine.
- To consider the suitability of using an ultrasonic washing machine for estimating TBAP and to evaluate the optimal working conditions through spike-and-recovery assessment.
- To verify the application of the proposed method to actual river water samples during base flows and high flows.

## Samples and methods

### Samples

The river water samples for this study were collected from the following three rivers in Aichi Prefecture, Japan: Toyo River, Asakura River, and Umeda River. These rivers were chosen because of their different land-use patterns (Table 1), which would result in different concentrations of P. Umeda River is in an agricultural area, while Toyo River is in a forested area. Asakura River catchment is constituted of 46% of forested areas in the upstream and dominance of urban areas along the river’s watershed; thus, it was designated as an urban river. Water samples were collected from these three rivers in June, September, and October 2020. On June 8, we collected 2 L of water samples from all three rivers for spike-and-recovery assessment of the proposed method. In September and October, to verify the application of the proposed method under various flow conditions, we collected 100 L of water samples from each river at each time. High-flow samples were collected from Umeda River at the beginning, in the middle, and at the end of a storm event on September 7–8, while base-flow samples were collected from all three rivers when there was little or no rain on October 7. The river water was collected in 20-L containers that were rinsed with analytical-grade deionized water. All samples were immediately transported to the laboratory.

**Table 1:** Land-use patterns of the rivers examined

River name	Land use			
	Agriculture	Urban	Forest	Others
Toyo	12	6	76	6
Asakura	7	43	46	4
Umeda	60	26	9	5

The filtrates of river water samples were separated from sediment by glass microfiber filters (Cytiva Whatman GF/F 47 mm; Global Life Sciences Technologies, Tokyo, Japan). The filtration was conducted as soon as the sample arrived at our laboratory to reduce storage effects on the dissolved P forms. The filters were then dried in an oven for 2 h at a temperature of 105 °C to determine SS concentrations. The sediment samples were separated from river water by continuous flow centrifugation (Himac CR22 G high-speed refrigerated centrifuge; R18C continuous rotor; Hitachi Koki, Ibaraki, Japan). The centrifugation was conducted at an average force of 20,000 × *g*, allowing for a discharge of 150 mL/min at 15,000 rpm. The river sediments collected after centrifugation were then dried in an oven at 40 °C for 72 h. Then, the oven-dried samples were sieved through a 0.149-mm-mesh screen to remove larger particles, such as plant fibers. All of the samples were placed in a sealed bag and stored at 4 °C until further analysis.

The sediments used for spike-and-recovery assessment were NIES CRM No. 31 (NIES Standard sediment 2012), which was purchased from the National Institute for Environmental Studies, Japan. NIES CRM No. 31 is the only freshwater reference material that the P concentration is certified so far by various analytical methods. We used this standard sediment adding to river water filtrates to create samples containing different P sediment concentrations for spike-and-recovery assessment. A sample matrix with the SS concentrations of 10, 50, and 300 mg/L was created for each of the three rivers.

### Extraction of bioavailable phosphorus

We are proposing a new extraction method involving an ultrasonic washing machine for routine laboratory analysis of TBAP in raw river water. In the proposed method, 19 mL of river water sample was placed in a 50-mL centrifuge vial with 1 mL of 2 M NaOH as the extractant to produce a concentration of 0.1 M NaOH in the final volume. The centrifuge tubes were then immersed inside the ultrasonic washing machine (Ultrasonic Cleaner Mus-10D, EYELA, Tokyo, Japan) for ultrasonic treatment. The water level in these machines had to be kept at the same levels as the solutions in the extraction vials. The extractions were then centrifuged at 1000 rpm (Table-top centrifuge model 5100 with an RS-4 universal swing rotor, Kubota, Tokyo, Japan) for 5 min. The clear supernatant of NaOH extract in each vial was collected for P measurement. After collecting the clear supernatant, 20 mL of 0.1 M NaOH was added to the remaining sediment, and then the extraction was repeated to find out the most suitable extraction number and extraction time, similar to the extraction of PBAP from sediments (Ka Ling et al. 2021).

The proposed method was also examined for extracting DBAP from river water filtrates without repetition. The DBAP concentrations extracted by the proposed method were compared with those extracted by conventional methods such as Sharpley’s mechanically shaking extraction (1991) and Ngoc’s ultrasonic extraction (2017). For all the samples, each extraction was evaluated in triplicates.

### Spike-and-recovery assessment

Spike-and-recovery assessment was designed to check the detection level of the proposed method (Lipps et al. 2022). For routine analysis purpose, we prepared a matrix spike for each river water. The concentrations of spiking solutions were 10, 50, and 300 mg/L of SS for each of the three rivers. The TBAP concentration in each spiking solution was simultaneously measured by the proposed extraction method, and calculated by the sum of DBAP and PBAP concentrations. The spiking DBAP concentration was determined in each river water filtrate. The spiking PBAP concentration was assumed by multiplying the SS concentration by the certified P concentration. The recovery was assessed by comparing the TBAP concentration obtained from extraction to the TBAP concentration obtained from calculation.

### Determination of phosphorus concentration

The concentrations of P extracted into the clear supernatant solution using the ultrasonic washing machine method were measured colorimetrically from the neutralized extracts using the molybdenum blue method. The intensity of the blue color, corresponding to the P concentration in the solution, was measured by flow injection analysis (Flow Injection Analyzer, FIA, OG-FI-300S, Ogawa & Co., Ltd., Hyogo, Japan). TP and DP were measured in the digested raw river water and the river water filtrates, respectively. In this case, digestion was performed using potassium peroxodisulfate (K_2_S_2_O_8_) in an autoclave at 120 °C for 30 min to convert all the P forms into soluble reactive phosphorus (SRP), which was detectable using the analyzer. PP was then determined as the difference between TP and DP. The non-BAP was determined as the difference between TP and TBAP. For the conventional method, TBAP was calculated by the sum of DBAP and PBAP. For the proposed method, TBAP was quantified directly using raw river water without the separation of sediment from water. All concentrations reported were obtained by means of triplicate analysis.

### Statistical analysis

A two-way analysis of variance (ANOVA) test and *t*-tests were used to compare the concentrations of extracted P using the proposed method and the conventional method. Linear regression analysis was also performed to address the similarities between the extracted P concentrations from both methods. Under the assumption that there were no significant differences between the methods, the significance was set to 5% to obtain more accurate results.

## Results and discussions

### Concentration of dissolved BAP

**Fig. 1:**
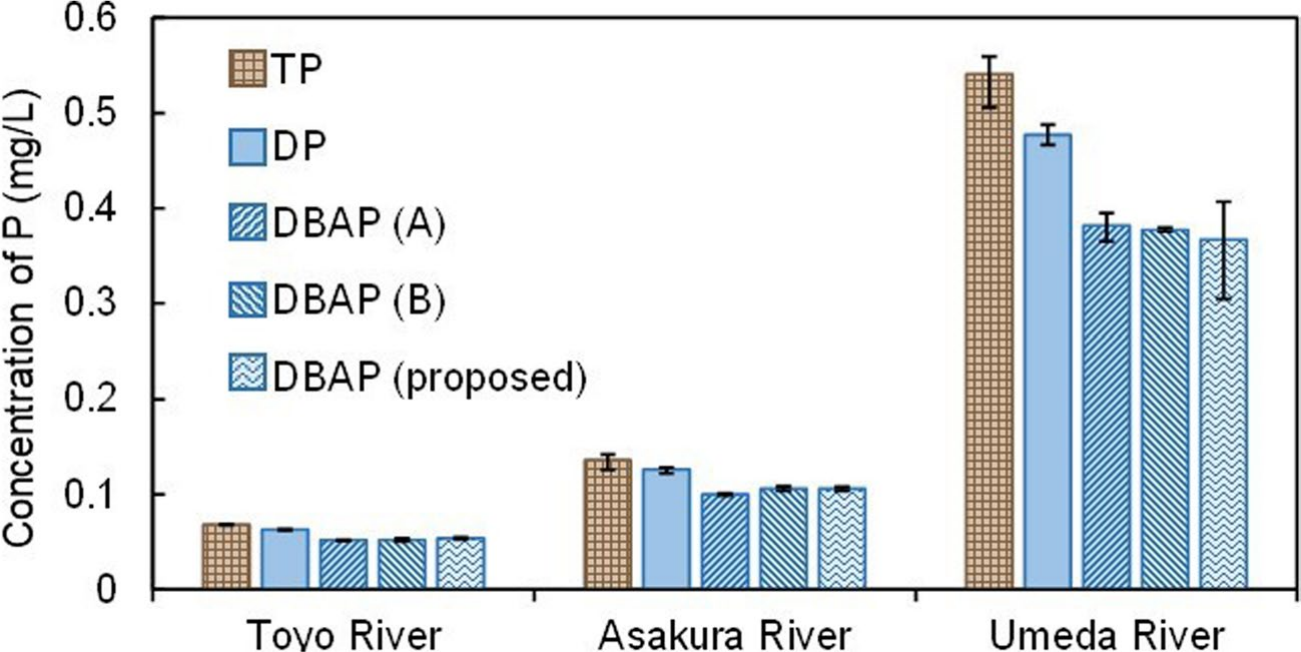
The concentrations of TP, DP, and DBAP extracted by conventional methods (A: Sharpley’s mechanically shaking extraction; B: Ngoc’s ultrasonic extraction) and the proposed method. Data are presented as means of triplicates ± SEM

DBAP was extracted using NaOH as a solvent in an ultrasonic washing machine. The results obtained from the proposed method were then compared with those from conventional methods, such as Sharpley’s mechanically shaking extraction and Ngoc’s ultrasonic extraction. In this experiment, we used water samples collected from Toyo, Asakura, and Umeda Rivers on June 8. The concentrations of DBAP for each river were evaluated with the concentrations of TP and DP (Fig. 1). Among the three rivers, the forested Toyo River was determined to have the lowest TP concentration, which was 0.068 mg/L, whereas the agricultural Umeda River had the highest TP concentration, which was 0.541 mg/L, approximately 9 times higher than that of Toyo River. The TP concentration in the Asakura River was 0.136 mg/L. Since the samples used in this experiment consisted of river water collected during base flow, DP accounted for most of the TP in all the river water samples. Additionally, SRP, which is an inorganic form of P, accounted for more than 90% of the DP. DBAP in Toyo and Asakura Rivers accounted for approximately 79% of the TP, while in Umeda River, it accounted for approximately 68% of the TP. The DBAP extracted with 0.1 M NaOH was lower than that of the DP digested using the potassium peroxodisulfate method. This indicated that some forms of P in the DP could not be hydrolyzed in a reactive form by NaOH. In other words, some forms of P in the DP were implied non-bioavailable. No significant difference was observed between the DBAP concentrations extracted by the proposed and conventional methods. Thus, the simultaneous multisample ultrasonic extraction method could extract DBAP as same as the conventional methods.

### Spike-and-recovery assessment

In this experiment, SS was added to river water filtrates to produce river water with different SS concentrations. Spike-and-recovery assessment was then conducted to examine the extraction performance of BAP using the simultaneous ultrasonic multisample extraction method. The concentrations of DBAP in the river water filtrates and PBAP in the SS were determined in advance. The target samples for this experiment were taken from Toyo, Asakura, and Umeda Rivers. The water samples were prepared with SS concentrations of 10, 50, and 300 mg/L.

**Fig. 2:**
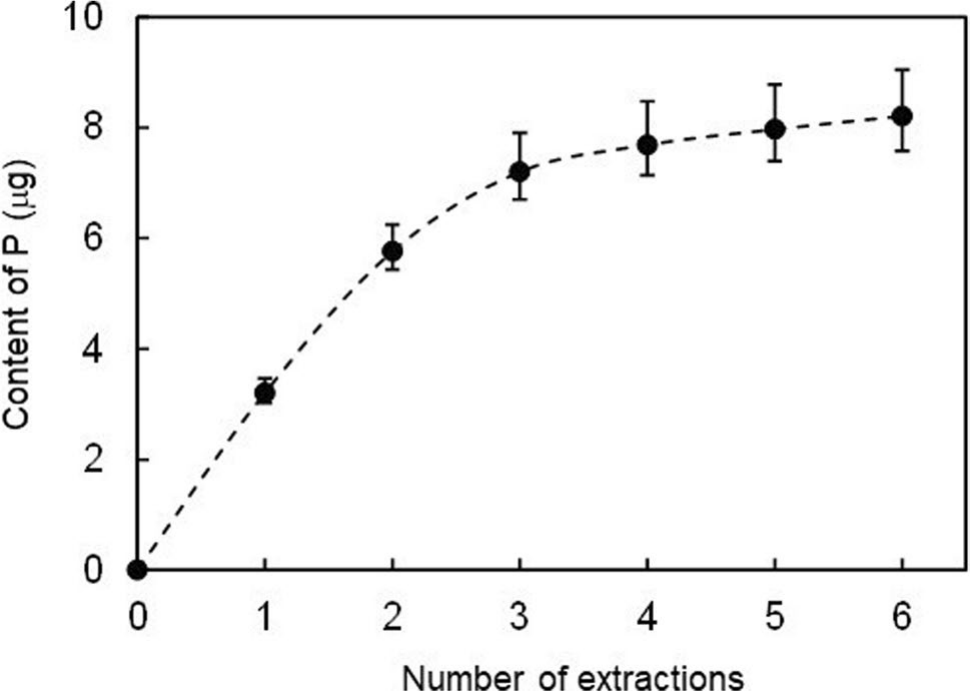
The cumulative TBAP content extracted as the number of repeated extractions increased. The extraction time was set up for 1 min. Data are presented as means of triplicates ± SEM

**Fig. 3:**
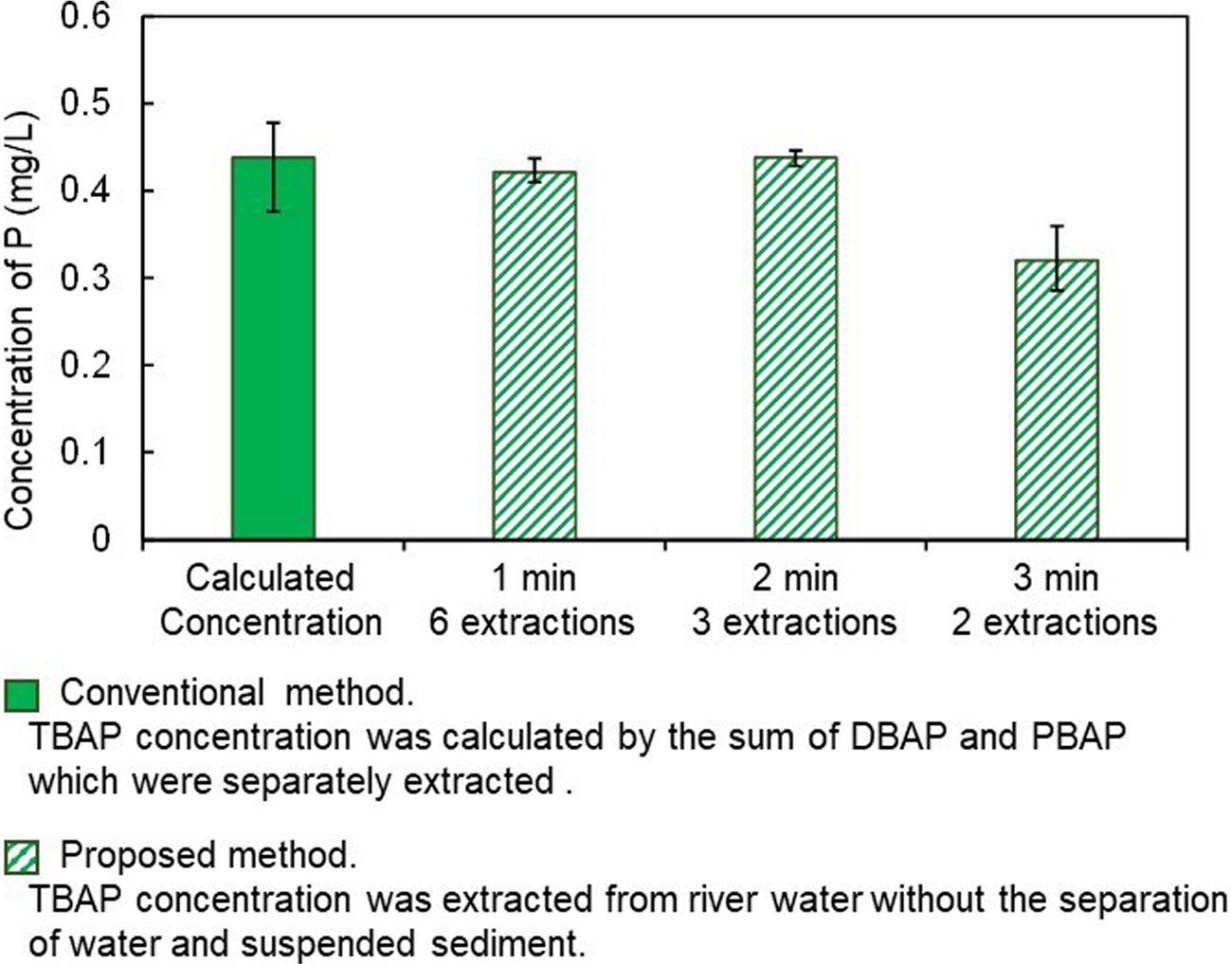
The concentrations of TBAP with varying extraction conditions. Data are presented as means of triplicates ± SEM

First, the number of extractions and the extraction time were considered. The experiment was conducted using water from Umeda River, where the SS concentration was 300 mg/L. Based on the finding of the extraction time for PBAP measurement (Ka Ling et al. 2021), the extraction time of 1 min was set up to find out the suitable extraction number for TBAP measurement. The extracted TBAP was accumulated when the number of repeated extractions increased (Fig. 2). As the number of repeated extractions increased, more P was extracted. When the number of extractions increased to 6, the concentration of extracted TBAP was consistent with the calculated value. However, after the third extraction, the extracted P content for each repeated extraction decreased, and the cumulative P concentration leveled off (Fig. 2). Therefore, we considered that the three extractions could measure the same TBAP concentration as the six extractions if the extraction time was increased from 1 to 2 min. It was found that the TBAP concentrations for the extraction time of 2 min and three extractions were consistent with the calculated concentration more than those for the extraction time of 1 min and six extractions (Fig. 3). Furthermore, we tested lessening the number of extractions to 2 and increasing the extraction time to 3 min, but the concentration of TBAP was lower than the calculated value (Fig. 3). These results indicated that the number of repeated extractions should be at least three to maintain the efficiency of extraction.

**Fig. 4:**
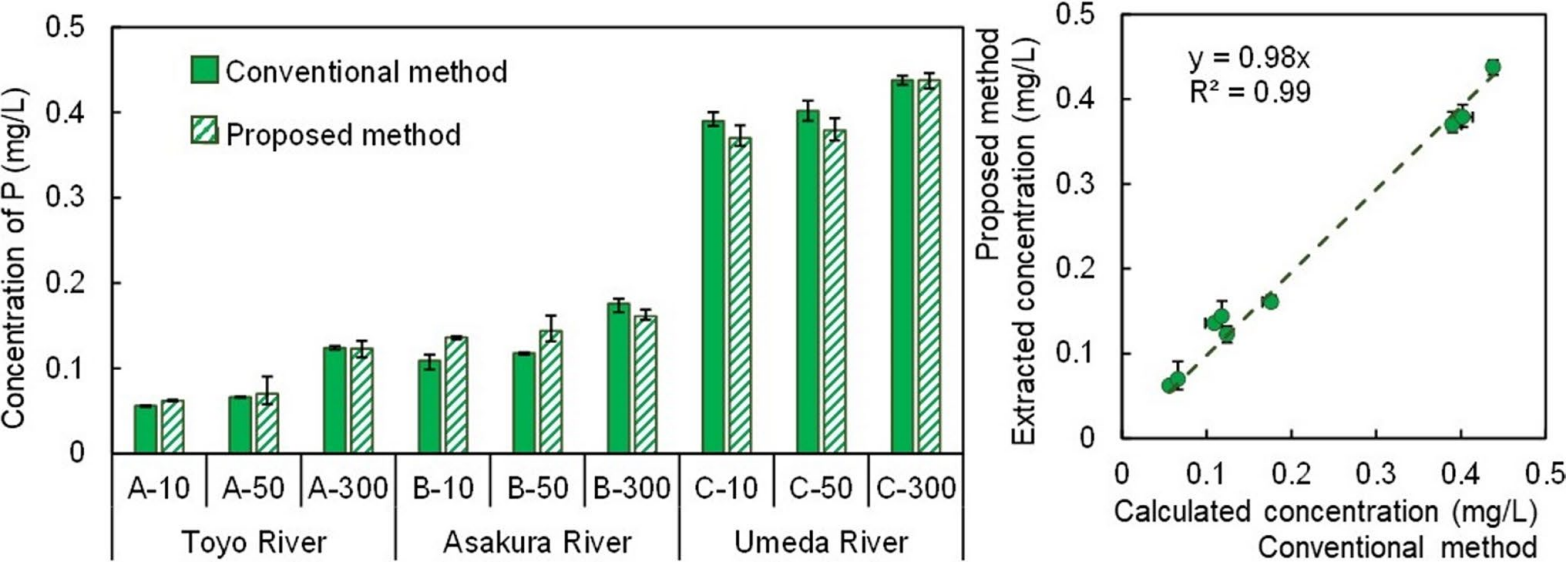
(left) The concentrations of TBAP in spike-and-recovery assessment. The conventional method calculated TBAP by the sum of DBAP in river water and PBAP in certified sediment, while the proposed method extracted TBAP from the spiking solution. (right) Correlation between the calculated concentration and the extracted concentration of TBAP. Data are presented as means of triplicates ± SEM

Next, the concentrations of TBAP (Fig. 4) in the prepared spiking river water samples were measured using the proposed TBAP extraction method. The extracted concentrations were detected from 0.056 to 0.438 mg/L of TBAP (Fig. 4, left). When the extracted concentrations of the three samples were compared with the TBAPs of the conventional methods, the extracted concentrations were found to account for more than 90% of those of the conventional methods. The correlation coefficient was 0.996, and the slope was 0.98 (Fig. 4, right). Both of these values were close to 1, indicating that the extracted concentrations were similar to the calculated concentrations from the conventional methods. Furthermore, linear regression analysis showed that *p* = 2.43 × 10^−8^, indicating that there was no significant difference between the experimental and calculated values. Therefore, an extraction time of 2 min and 3 extractions were found to be the most suitable conditions for the extraction of TBAP from river water through simultaneous multisample ultrasonic extraction.

### Adaptation experiment

#### Adaptation of the extraction method

**Fig. 5:**
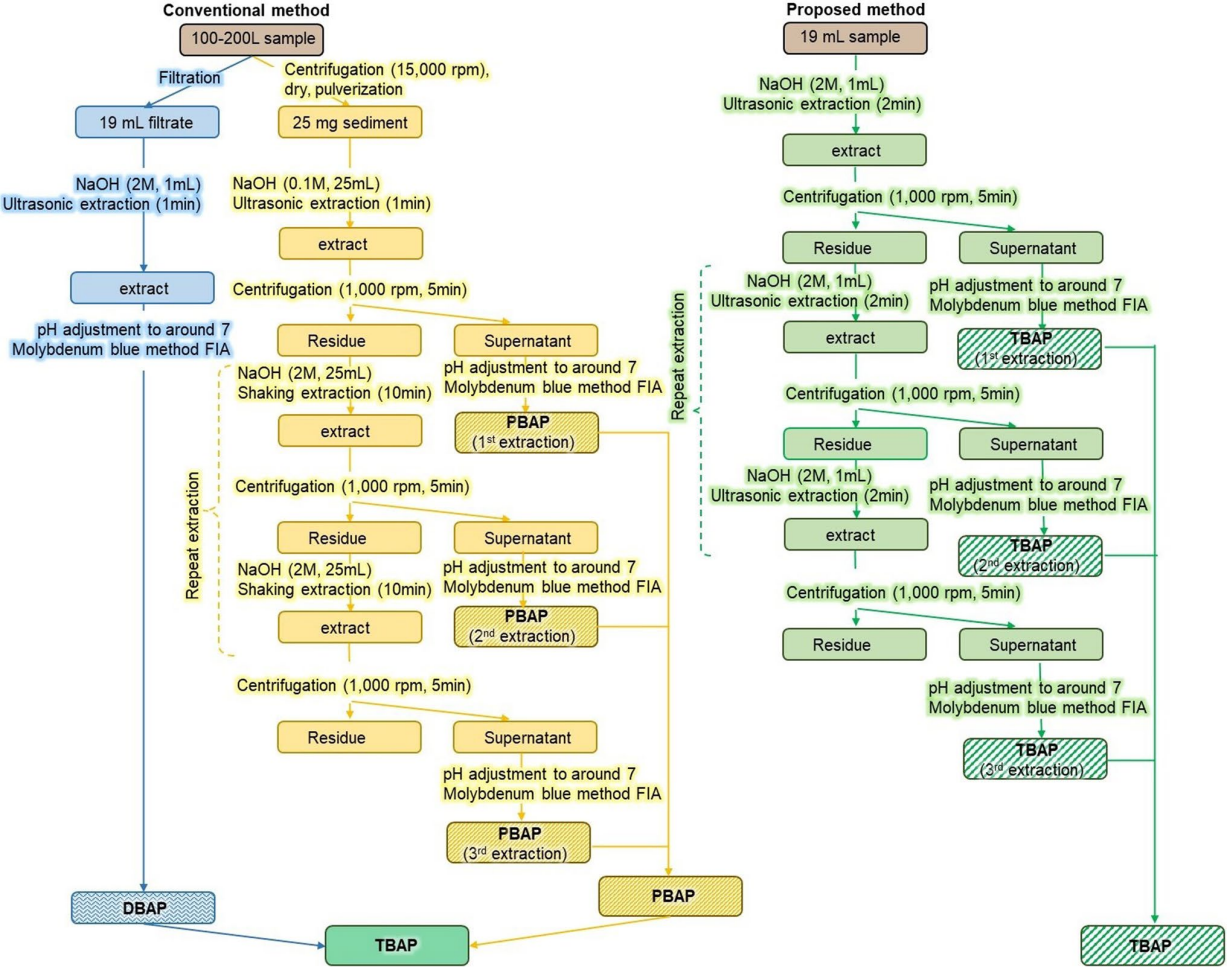
Flow chart for the conventional method and proposed method for TBAP analysis

In this experiment, we used 100 L of river water samples, which were collected from Toyo, Asakura, and Umeda Rivers during high flow in September, and base flow in October. The TBAP values of all the rivers were quantified using the conventional method and the proposed method to examine the practicality of the simultaneous multisample ultrasonic extraction method and its adaptability for actual river water analysis. In the conventional method, the TBAP concentration in river water is calculated as the sum of DBAP and PBAP. The DBAP concentration was determined by extraction of river water filtrate, while the PBAP concentration was extracted from the suspended solids which were separated from the river water by continuous flow centrifugation. In contrast, in the proposed method, the TBAP was extracted directly from the river water. Both of the methods for TBAP measurement were step-by-step illustrated (Fig. 5).

#### Variation in suspended sediments and phosphorus fractions

**Fig. 6:**
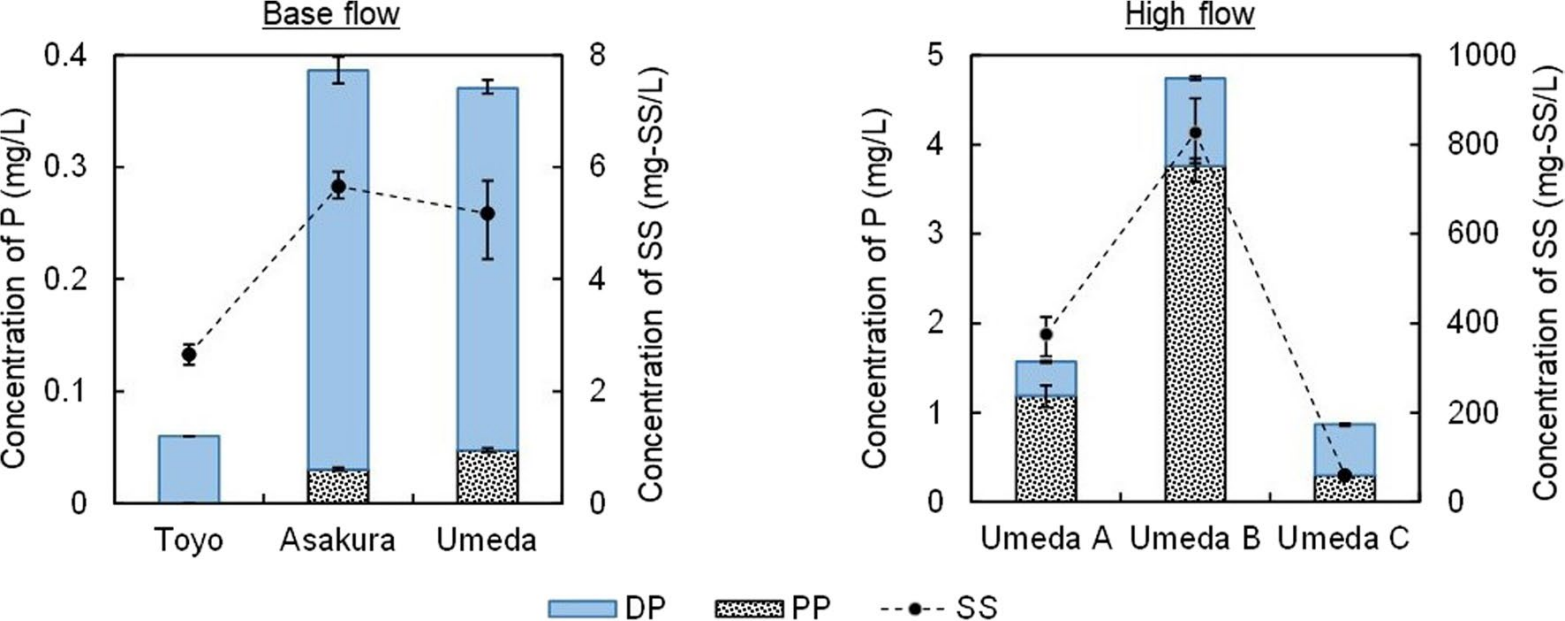
Concentrations of DP, PP, and SS during base flows (left) and high flows (right). Data are presented as means of triplicates ± SEM

The SS, TP, DP, and PP concentrations under base flow and high flow were investigated (Fig. 6). During base flow, the concentrations of SS in Toyo, Asakura, and Umeda Rivers were 2.7, 5.6, and 5.2 mg/L, respectively. Toyo River was determined to have the lowest SS concentration, whereas Asakura River had the highest, but the concentration in Asakura River was almost the same as that of Umeda River. The TP concentration in Toyo River was the lowest at 0.060 mg/L, that in Umeda River was 0.371 mg/L, and that in Asakura River was the highest at 0.386 mg/L. The DP concentration in Toyo River was 0.060 mg/L, which was approximately the same as TP. This indicated that the TP in Toyo River was mainly composed of dissolved forms of P. The DP concentrations in Umeda and Asakura Rivers were determined to be 0.323 and 0.356 mg/L, respectively, indicating that approximately 90% of the TP was in a dissolved form.

During storm events, water samples were collected from the Umeda River 3 times. The SS concentrations were as follows: A: 375.7 mg/L, B: 826.9 mg/L, and C: 59.8 mg/L. The SS concentration was the highest in the Umeda River B sample because the water was collected during high flow. The TP concentration during high flow was 4.741 mg/L. When the flow rate increased from the base flow, the TP was 1.574 mg/L; however, when the flow rate decreased, it dropped to 0.871 mg/L. When the SS increased, the TP concentration also increased. During high flow, DP was 0.982 mg/L, which accounted for only approximately 20% of the TP. The remaining 80% was in the particulate form, indicating a strong relationship between SS and TP.

Comparing the results of the water quality survey during base flow and high flow, it was determined that the SS concentration during high flow was greater than that during base flow. Furthermore, as the flow rate increased with rainfall, the SS concentration also increased. This might be attributed to the inflow of soil from riverbanks; additionally, sediments on river bottoms were swept up by the flow. The TP concentration during high flow was higher than that during base flow. The TP was mainly DP during base flow, whereas it consisted of 80% of PP during high flow. As a result, the SS and the P fractions differed greatly during base flow and high flow.

#### Variation in bioavailable phosphorus

**Fig. 7:**
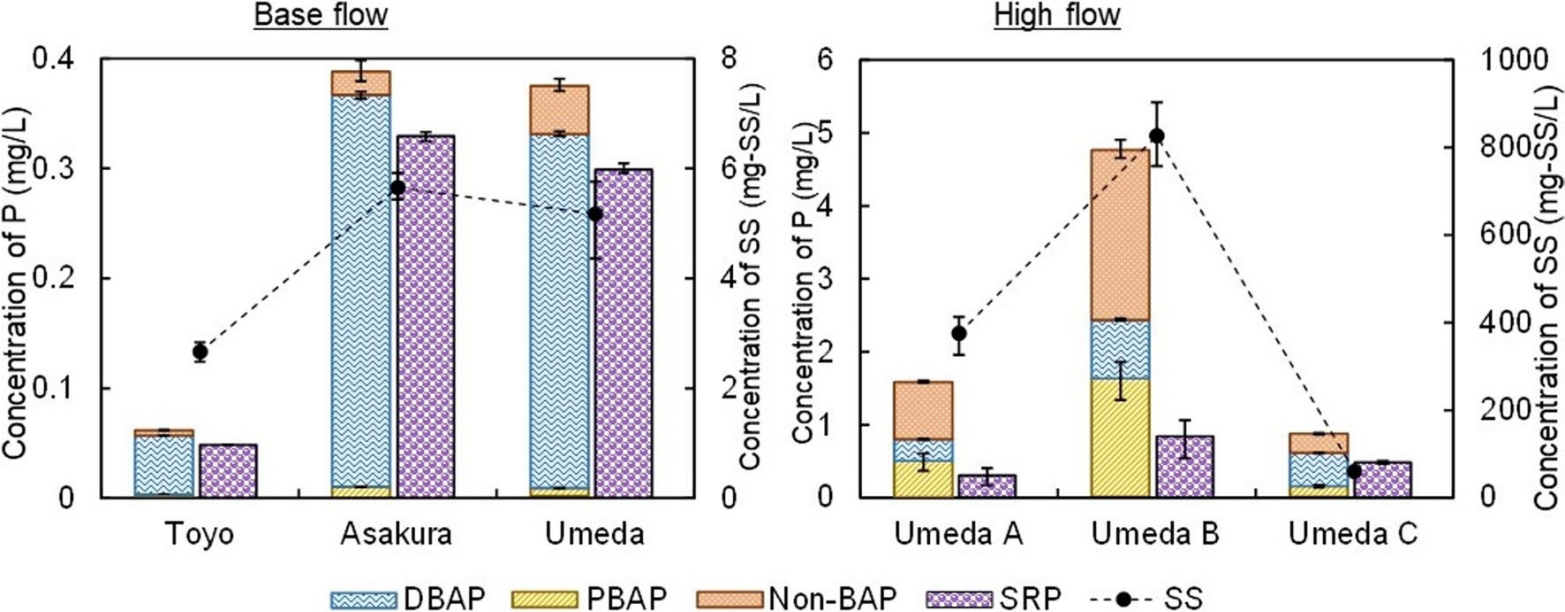
Concentrations of BAP and SS during base flows (left) and high flows (right). Data are presented as means of triplicates ± SEM

Based on the procedure for the conventional method of TBAP analysis (Fig. 5), the sum of the concentrations of DBAP and PBAP was considered as the TBAP (Fig. 7). The concentrations of DBAP under base flow and high flow (Fig. 7) were found to be lower than those of DP measured using the standard method (Fig. 6), i.e., the potassium peroxodisulfate digestion method, but were higher than those of SRP (Fig. 7). This result showed that the DP included P that could not be directly utilized by algae. These forms of P are considered to be a part of the dissolved organic P fraction (Pacini and Gächter 1999).

The TBAP concentrations in Umeda River were higher during high flow than during base flow. In the case of base flow, TBAP was mainly in dissolved forms, but in the case of the high flow, PBAP accounted for a larger proportion. Additionally, approximately 90% of the TP was bioavailable during base flow, whereas only approximately 50% of the TP could be utilized by algae during high flow. Therefore, BAP should be a parameter considered in environmental standards since TP analysis alone is insufficient for the management of aquatic environments.

#### Adaptability of the study method

**Fig. 8:**
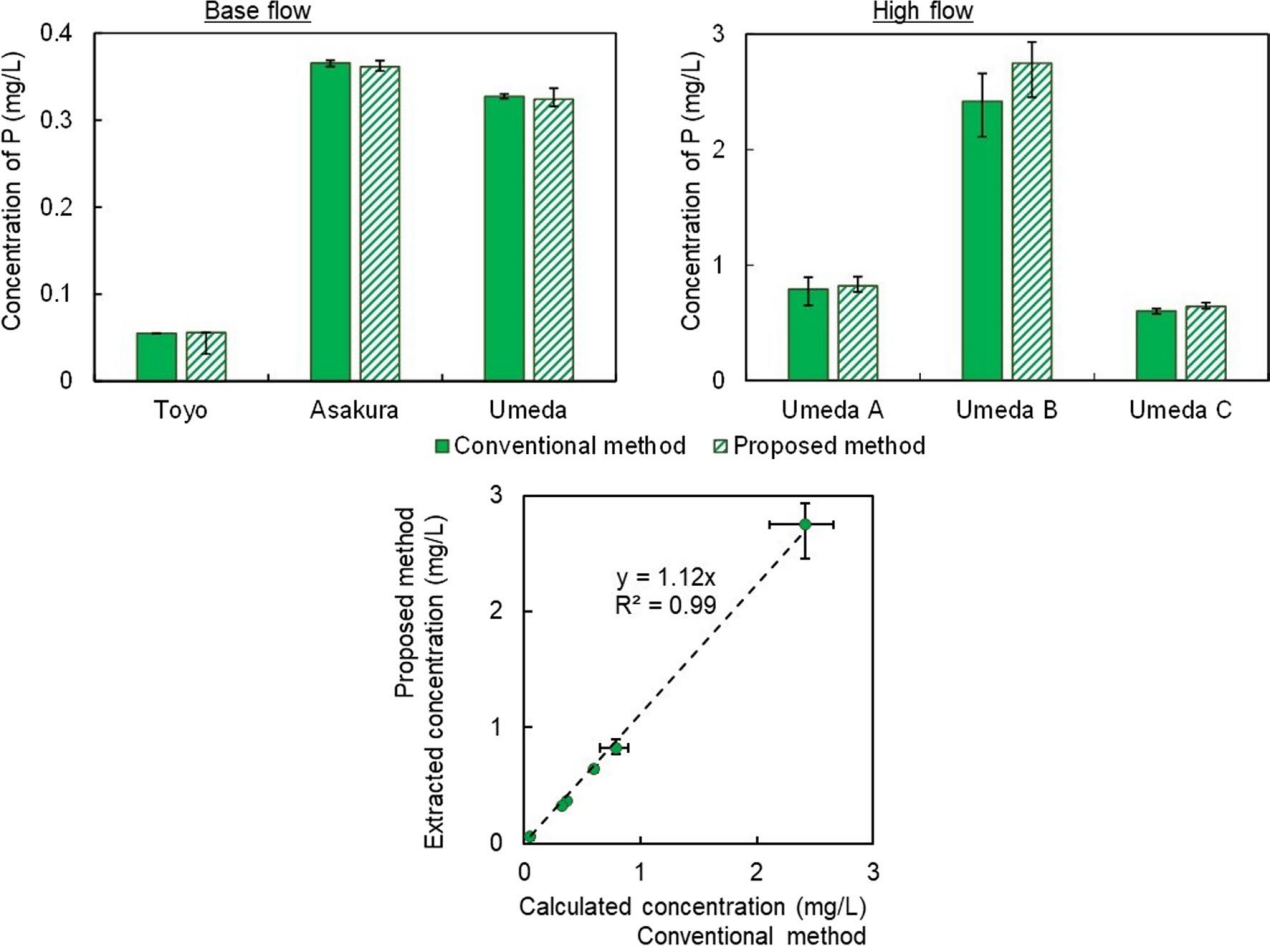
(top) The concentrations of TBAP in rivers. The conventional method calculated TBAP by the sum of DBAP in river water filtrates and PBAP in river sediments, while the proposed method extracted TBAP from raw river water. (bottom) Correlation between the calculated concentration and the extracted concentration of TBAP. Data are presented as means of triplicates ± SEM

The TBAP concentrations under base flow and high flow were investigated (Fig. 8). The TBAP concentrations ranged from 0.056 to 2.748 mg/L. The TBAP concentrations were compared with the TBAP concentrations from the conventional method, and the extracted concentration was more than 95% of that of the conventional method. Correlation analysis was conducted to study the relationship between the conventional method and the proposed method. The results showed that the correlation coefficient was 0.99, and the slope was 1.1, which was close to 1 for both methods. Therefore, it was confirmed that the concentration extracted by the proposed method was approximately equal to that of the conventional method. Furthermore, the results of the test for significant difference by linear regression showed that *p* = 2.35 × 10^−11^; thus, no significant difference was observed between the conventional method and the proposed method. Thus, the proposed method for TBAP analysis was capable of extracting all BAP directly from river water.

#### Benefits of the proposed method

The proposed extraction method for simultaneous ultrasonic multisample analysis can be used to extract TBAP directly from river water. Thus, the separation of sediment from river water becomes unnecessary. This shortened measurement time relative to the conventional method, which requires more than 72 h to complete.

Furthermore, in the ultrasonic multisample extraction method, TBAP is extracted using an ultrasonic washing machine, which is a common piece of laboratory equipment that can be obtained at a reasonable price. Additionally, an ultrasonic washing machine can be used to extract multiple samples simultaneously during a single run, reducing the time required for conducting the experiment. Therefore, the proposed method is considered to be more versatile, economical, and convenient than the conventional TBAP method.

The proposed method can shorten measurement time compared with the conventional TP method because it does not require high-heat digestion in an autoclave. However, the proposed method has a drawback in terms of the workload required during the experiment. The extraction procedure in the proposed method has to be repeated 3 times, which constitutes a greater workload than that of the TP method.

## Conclusion

According to our findings, BAP varied significantly between base flow and high flow. During base flow, BAP was mainly in a dissolved form, while during high flow, the particulate form of BAP accounted for a larger proportion. Furthermore, during base flow, a strong correlation was found between the TP, which has been used as a standard parameter for eutrophication monitoring, and the BAP, which is defined actually available to the organisms. In contrast, during high flow, a significant but not strong relationship was observed between BAP and TP. It might be due to the decreased bioavailability of PP at high flow. On the other hand, the concentrations of DBAP extracted by the proposed method were lower than those of DP digested by the persulfate method; however, they were higher than those of SRP, implying that DP contained a small proportion of P that might be not biological. Therefore, PBAP has been often measured separately from DBAP, and the sum of them has been used to estimate TBAP in rivers.

In this study, we examined a simultaneous multisample ultrasonic extraction method to fasten the extraction DBAP and PBAP. The extracted concentrations were similar to those obtained by using conventional methods, such as Sharpley’s mechanically shaking extraction and Ngoc’s ultrasonic extraction. Then, we proposed an extraction procedure for simultaneous ultrasonic multisample analysis of TBAP in rivers without the separation of DBAP from PBAP. In the spike-and-recovery assessment, the P concentrations obtained using 2-min extraction times and three repeated extractions were consistent with the spike calculations. Furthermore, the results of TBAP extraction under these conditions from river water containing spiked P showed no differences between the experimental and calculated values, and the statistical analysis also showed no significant difference. The spiked TBAP values were from 0.056 to 0.438 mg/L. Besides, the applicability of the extraction method for the simultaneous multisample ultrasonic extraction of BAP from real river water was investigated. The concentrations of TBAP extracted from river water ranged from 0.056 to 2.748 mg/L. The concentrations of TBAP extracted by the proposed method were found to be approximately the same as those in the conventional method, and no statistically significant difference was observed. It suggested that the separation of DBAP from PBAP was unnecessary. TBAP could be directly measured in river water which has TBAP in the range of 0.056 to 2.748 mg/L. The proposed method is much simpler than the conventional methods, and it will allow us to more easily evaluate BAP, to faster evaluate eutrophication risks in rivers.

## Data Availability

The data supporting the findings of this study are available within the manuscript.

## Abbreviations

SS: Suspended sediment
TP: Total phosphorus
DP: Dissolved phosphorus
PP: Particulate phosphorus
BAP: Bioavailable phosphorus
TBAP: Total bioavailable phosphorus
DBAP: Dissolved bioavailable phosphorus
PBAP: Particulate bioavailable phosphorus
